# Mental health and music engagement: review, framework, and guidelines for future studies

**DOI:** 10.1038/s41398-021-01483-8

**Published:** 2021-06-22

**Authors:** Daniel E. Gustavson, Peyton L. Coleman, John R. Iversen, Hermine H. Maes, Reyna L. Gordon, Miriam D. Lense

**Affiliations:** 1 Department of Medicine, Vanderbilt University Medical Center, Nashville, TN USA; 2 Vanderbilt Genetics Institute, Vanderbilt University Medical Center, Nashville, TN USA; 3 Department of Otolaryngology – Head & Neck Surgery, Vanderbilt University Medical Center, Nashville, TN USA; 4 Swartz Center for Computational Neuroscience, Institute for Neural Computation, University of California, San Diego, La Jolla, CA USA; 5 Department of Human and Molecular Genetics, Virginia Institute for Psychiatric and Behavioral Genetics, Virginia Commonwealth University, Richmond, VA USA; 6 Department of Psychiatry, Virginia Institute for Psychiatric and Behavioral Genetics, Virginia Commonwealth University, Richmond, VA USA; 7 Massey Cancer Center, Virginia Commonwealth University, Richmond, VA USA; 8 Vanderbilt Brain Institute, Vanderbilt University, Nashville, TN USA; 9 The Curb Center, Vanderbilt University, Nashville, TN USA

**Keywords:** Psychiatric disorders, Medical genetics

## Abstract

Is engaging with music good for your mental health? This question has long been the topic of empirical clinical and nonclinical investigations, with studies indicating positive associations between music engagement and quality of life, reduced depression or anxiety symptoms, and less frequent substance use. However, many earlier investigations were limited by small populations and methodological limitations, and it has also been suggested that aspects of music engagement may even be associated with worse mental health outcomes. The purpose of this scoping review is first to summarize the existing state of music engagement and mental health studies, identifying their strengths and weaknesses. We focus on broad domains of mental health diagnoses including internalizing psychopathology (e.g., depression and anxiety symptoms and diagnoses), externalizing psychopathology (e.g., substance use), and thought disorders (e.g., schizophrenia). Second, we propose a theoretical model to inform future work that describes the importance of simultaneously considering music-mental health associations at the levels of (1) correlated genetic and/or environmental influences vs. (bi)directional associations, (2) interactions with genetic risk factors, (3) treatment efficacy, and (4) mediation through brain structure and function. Finally, we describe how recent advances in large-scale data collection, including genetic, neuroimaging, and electronic health record studies, allow for a more rigorous examination of these associations that can also elucidate their neurobiological substrates.

## Introduction

Music engagement, including passive listening and active music-making (singing, instrument playing), impacts socio-emotional development across the lifespan (e.g., socialization, personal/cultural identity, mood regulation, etc.), and is tightly linked with many cognitive and personality traits [1–3]. A growing literature also demonstrates beneficial associations between music engagement and quality of life, well-being, prosocial behavior, social connectedness, and emotional competence [4–8]. Despite these advances linking engagement with music to many wellness characteristics, we have a limited understanding of how music engagement directly and indirectly contributes to mental health, including at the trait-level (e.g., depression and anxiety symptoms, substance use behaviors), clinical diagnoses (e.g., associations with major depressive disorder (MDD) or substance use disorder (SUD) diagnoses), or as a treatment. Our goals in this scoping review are to (1) describe the state of music engagement research regarding its associations with mental health outcomes, (2) introduce a theoretical framework for future studies that highlight the contribution of genetic and environmental influences (and their interplay) that may give rise to these associations, and (3) illustrate some approaches that will help us more clearly elucidate the genetic/environmental and neural underpinnings of these associations.

### Scope of the article

People interact with music in a wide variety of ways, with the concept of “musicality” broadly including music engagement, music perception and production abilities, and music training [9]. Table 1 illustrates the breadth of music phenotypes and example assessment measures. Research into music and mental health typically focuses on measures of music engagement, including passive (e.g., listening to music for pleasure or as a part of an intervention) and active music engagement (e.g., playing an instrument or singing; group music-making), both of which can be assessed using a variety of objective and subjective measures. We focus primarily on music engagement in the current paper but acknowledge it will also be important to examine how mental health traits relate to other aspects of musicality as well (e.g., perception and production abilities).

**Table 1:** Diversity of the musical phenotype and example assessment measures.

	Active engagement/Musical training	Emotional response to music	Social aspects	Dance/Music during physical activity	Musical seeking	Music for coping	Musical expression	Music-making ability	Tonal perception	Rhythmic production	Rhythmic perception
**Self-report**											
Goldsmiths Music Sophistication Index (Gold-MSI)	✓	✓					✓				
Music Engagement Questionnaire (MEQ)		✓	✓		✓						
Barcelona Music Reward Questionnaire		✓	✓	✓	✓	✓					
Music Use Questionnaire (MUSE)	✓	✓	✓	✓							
Adaptive Functions of Music Listening Scale		✓				✓					
Healthy-Unhealthy Music Scale (HUMS)		✓	✓			✓					
Emotion Regulation Strategies for Artistic Creative Achievement		✓				✓					
Motivation and Engagement Scale - Music (MES-M)	✓				✓	✓					
**Informant-report**											
Beech Brook Music Therapy Assessment		✓	✓				✓	✓			
Music Therapy Rating Scale		✓					✓				
Music Engagement Questionnaire (MusEQ)^a^		✓			✓		✓	✓			
**Objective measures**											
Battery for the Assessment of Auditory Sensorimotor and Timing Abilities (BAASTA)										✓	✓
Montral Battery of Evaluation of Amusia (MBEA)									✓		✓
Advanced Measures of Music Audiation (AMMA)									✓		✓
AIRS Test Battery of Singing Skills (ATBSS)								✓		✓	
Profile of Music Perception Skills (PROMS)							✓		✓		✓
Beat Alignment Test (BAT)										✓	✓
Harvard Beat Assessment Test (H-BAT)										✓	✓
Swedish Musical Discrimination Test (SMDT)									✓		✓

Active engagement/Music Training = music making for fun; singing, playing, or making music inside or outside of formal training/lessons; performing. Emotional response to music = music-evoked emotion, affective reactions to music. Social aspects = social connection via music, music as identity. Dance/Music during physical activity = dance experiences, music listening during physical activity. Musical seeking = music listening habits, motivation to engage in music. Music for coping = music listening for mood regulation or stress relief, meditation with music. Musical expression = ability to make music expressively, putting emotion into musical performance, communication using music. Music-making ability = singing ability, instrumental skill, vocal range. Tonal perception = melodic, pitch, or harmony discrimination. Rhythmic production = ability to tap to a beat, ability to repeat rhythms. Rhythmic perception = rhythmic discrimination, metrical perception.

^a^The MusEQ can be either self-report or informant-report.

Our scoping review and theoretical framework incorporate existing theoretical and mechanistic explanations for how music engagement relates to mental health. From a psychological perspective, studies have proposed that music engagement can be used as a tool for encouraging self-expression, developing emotion regulation and coping skills, and building community [10, 11]. From a physiological perspective, music engagement modulates arousal levels including impacts on heart rate, electrodermal activity, and cortisol [12, 13]. These effects may be driven in part by physical aspects of music (e.g., tempo) or rhythmic movements involved in making or listening to music, which impact central nervous system functioning (e.g., leading to changes in autonomic activity) [14], as well as by personality and contextual factors (e.g., shared social experiences) [15]. Musical experiences also impact neurochemical processes involved in reward processing [10, 13, 14, 16–18], which are also implicated in mental health disorders (e.g., substance use; depression). Thus, an overarching framework for studying music-mental health associations should integrate the psychological, physiological, and neurochemical aspects of these potential associations. We propose expanding this scope further through consideration of genetic and environmental risk factors, which may give rise to (and/or interact with) other factors to impact health and well-being.

Regarding mental health, it is important to recognize the hierarchical structure of psychopathology [19, 20]. Common psychological disorders share many features and cluster into internalizing (e.g., MDD, generalized anxiety disorder (GAD), posttraumatic stress disorder (PTSD)), externalizing (e.g., SUDs, conduct disorder), and thought disorders (e.g., bipolar disorder, schizophrenia), with common variance shared even across these domains [20]. These higher-order constructs tend to explain much of the comorbidity among individual disorders, and have helped researchers characterize associations between psychopathology, cognition, and personality [21–23]. We use this hierarchical structure to organize our review. We first summarize the emerging literature on associations between music engagement and generalized well-being that provides promising evidence for associations between music engagement and mental health. Next, we summarize associations between music engagement and internalizing traits, externalizing traits/behaviors, and thought disorders, respectively. Within these sections, we critically consider the strengths and shortcomings of existing studies and how the latter may limit the conclusions drawn from this work.

**Fig. 1:**
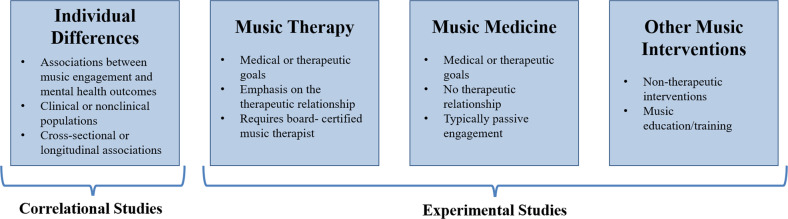
Example study designs in music-mental health research. Within experimental studies, music interventions can include passive musical activities (e.g., song listening, music and meditation, lyric discussion, creating playlists) or active musical activities (e.g., creative methods, such as songwriting or improvisation and/or re-creative methods, such as song parody).

Our review considers both correlational and experimental studies (typically, intervention studies; see Fig. 1 for examples of study designs). We include not only studies that examine symptoms or diagnoses based on diagnostic interviews, but also those that assess quantitative variation (e.g., trait anxiety) in clinical and nonclinical populations. This is partly because individuals with clinical diagnoses may represent the extreme end of a spectrum of similar, sub-clinical, problems in the population, a view supported by evidence that genetic influences on diagnosed psychiatric disorders or DSM symptom counts are similar to those for trait-level symptoms in the general population [24, 25]. Music engagement may be related to this full continuum of mental health, including correlations with trait-level symptoms in nonclinical populations and alleviation of symptoms from clinical disorders. For example, work linking music engagement to subjective well-being speaks to potential avenues for mental health interventions in the population at large.

The goal of this scoping review was to integrate across related, but often disconnected, literatures in order to propose a comprehensive theoretical framework for advancing our understanding of music-mental health associations. For this reason, we did not conduct a fully systematic search or quality appraisal of documents. Rather, we first searched PubMed and Google Scholar for review articles and meta-analyses using broad search terms (e.g., “review” and “music” and [“anxiety” or “depression” or “substance use”]). Then, when drafting each section, we searched for additional papers that have been published more recently and/or were examples of higher-quality research in each domain. When giving examples, we emphasize the most recent and most well-powered empirical studies. We also conducted some targeted literature searches where reviews were not available (e.g., “music” and [“impulsivity” or “ADHD”]) using the same databases. Our subsequent framework is intended to contextualize diagnostic, symptom, and mechanistic findings more broadly within the scope of the genetic and environmental risk factors on psychopathology that give rise to these associations and (potentially) impact the efficacy of treatment efforts. As such, the framework incorporates evidence from review articles and meta-analyses from various literatures (e.g., music interventions for anxiety [26], depression [27]) in combination with experimental evidence of biological underpinnings of music engagement and the perspective provided by newly available methods for population-health approaches (i.e., complex trait genetics, gene–environment interactions).

### Music engagement and well-being

A growing body of studies report associations between music engagement and general indices of mental health, including increased well-being or emotional competence, lending support for the possibility that music engagement may also be associated with better specific mental health outcomes. In over 8000 Swedish twins, hours of music practice and self-reported music achievement were associated with better emotional competence [5]. Similarly, a meta-ethnography of 46 qualitative studies revealed that participation in music activities supported well-being through management of emotions, facilitation of self-development, providing respite from problems, and facilitating social connections [28]. In a sample of 1000 Australian adults, individuals who engaged with music, such as singing or dancing with others or attending concerts reported greater well-being vs. those who engaged in these experiences alone or did not engage. Other types of music engagement, such as playing an instrument or composing music were not associated with well-being in this sample [4]. Earlier in life, social music experiences (including song familiarity and synchronous movement to music) are associated with a variety of prosocial behaviors in infants and children [6], as well as positive affect [7]. Thus, this work provides some initial evidence that music engagement is associated with better general mental health outcomes in children and adults with some heterogeneity in findings depending on the specific type of music engagement.

### Music engagement and internalizing problems

MDD, GAD, and PTSD are the most frequently clustered aspects of internalizing psychopathology [19, 24, 29, 30]. Experimental studies provide evidence for the feasibility of music intervention efforts and their therapeutic benefits but are not yet rigorous enough to draw strong conclusions. The most severe limitations are small samples, the lack of appropriate control groups, few interventions with multiple sessions, and publications omitting necessary information regarding the intervention (e.g., intervention fidelity, inclusion/exclusion criteria, education status of intervention leader) [31–33]. Correlational studies, by contrast, suggest musicians are at greater risk for internalizing problems, but that they use music engagement as a tool to help manage these problems [34, 35].

### Experimental studies

Randomized controlled trials have revealed that music interventions (including both music therapies administered by board-certified music therapists and other music interventions) are associated with reduced depression, anxiety, and PTSD symptoms [26, 27, 33, 36]. A review of 28 studies reported that 26 revealed significantly reduced depression levels in music intervention groups compared to control groups, including the 9 studies which included active non-music intervention control groups (e.g., reading sessions, “conductive-behavior” psychotherapy, antidepressant drugs) [27]. A similar meta-analysis of 19 studies demonstrated that music listening is effective at decreasing self-reported anxiety in healthy individuals [26]. A review of music-based treatment studies related to PTSD revealed similar conclusions [36], though there were only four relevant studies. More recent studies confirm these findings [37–39], such as one randomized controlled trial that demonstrated reduced depression symptoms in older adults following musical improvisation exercises compared to an active control group (gentle gymnastic activities) [39].

This work is promising given that some studies have observed effects even when compared to traditional behavior therapies [40, 41]. However, there are relatively few studies directly comparing music interventions to traditional therapies. Some music interventions incorporate components of other therapeutic methods in their programs including dialectic or cognitive behavior therapies [42], but few directly compare how the inclusion of music *augments* traditional behavioral therapy. Still other non-music therapies incorporate music into their practice (e.g., background music in mindfulness therapies) [43, 44], but the specific contribution of music in these approaches is unclear. Thus, there is a great need for further systematic research relating music to traditional therapies to understand which components of music interventions act on the same mechanisms as traditional therapies (e.g., developing coping mechanisms and building community) and which bolster or synchronize with other approaches (e.g., by adding structure, reinforcement, predictability, and social context to traditional approaches).

Aside from comparison with other therapeutic approaches, an earlier review of 98 papers from psychiatric in-patient studies concluded that promising effects of music therapy were limited by small sample sizes and methodological shortcomings including lack of reporting of adverse events, exclusion criteria, possible confounders, and characteristics of patients lost to follow-up [33]. Other problems included inadequate reporting of information on the source population (e.g., selection of patients and proportion agreeing to take part in the study), the lack of masking of interviewers during post-test, and concealment of randomization. Nevertheless, there was some evidence that therapies with active music participation, structured sessions, and multiple sessions (i.e., four or more) improved mood, with all studies incorporating these characteristics reporting significant positive effects. However, most studies have focused on passive interventions, such as music listening [26, 27]. Active interventions (e.g., singing, improvising) have not been directly compared with passive interventions [27], so more work is needed to clarify whether therapeutic effects are indeed stronger with more engaging and active interventions.

### Correlational studies

Correlational studies have focused on the use of music in emotional self-regulation. Specifically, individuals high in neuroticism appear to use music to help regulate their emotions [34, 35], with beneficial effects of music engagement on emotion regulation and well-being driven by cognitive reappraisal [45]. Music listening may also moderate the association between neuroticism and depression in adolescents [46], consistent with a protective effect.

A series of recent studies have used validated self-reported instruments that directly assess how individuals use music activities as an emotion regulation strategy [47–50]. In adults, the use of music listening for anger regulation and anxiety regulation was positively associated with subjective well-being, psychological well-being, and social well-being [50]. In studies of adolescents and undergraduates, the use of music listening for entertainment was associated with fewer depression and anxiety symptoms [51]. “Healthy” music engagement in adolescents (i.e., using music for relaxation and connection with others) was also positively associated with happiness and school satisfaction [49]. However, the use of music listening for emotional discharge was also associated with greater depression, anxiety, and stress symptoms [51], and “unhealthy” music engagement (e.g., ‘hiding’ in music to block others out) was associated with lower well-being, happiness, school satisfaction, and greater depression and rumination [49]. Other work has highlighted the role of valence in these associations, with individuals who listen to happier music when they are in a bad mood reporting stronger ability for music to influence their mood than those who listen to sad music while in a negative mood [52, 53].

This work highlights the importance of considering individuals’ motivations for engaging with music in examining associations with well-being and mental health, and are consistent with the idea that individuals already experiencing depression, anxiety, and stress use music as a therapeutic tool to manage their emotions, with some strategies being more effective than others. Of course, these correlational effects may not necessarily reflect causal associations, but could be due to bidirectional influences, as suggested by claims that musicians may be at higher risk for internalizing problems [54–56]. It is also necessary to consider demographic and socioeconomic factors in these associations [57], for example, because arts engagement may be more strongly associated with self-esteem in those with higher education [58].

It is also necessary to clarify if musicians (professional and/or nonprofessional) represent an already high-risk group for internalizing problems. In one large study conducted in Norway (*N* = 6372), professional musicians were higher in neuroticism than the general population [56]. Another study of musician cases (*N* = 9803) vs. controls (*N* = 49,015) identified in a US-based research database through text-mining of medical records found that musicians are at greater risk of MDD (Odds ratio [OR] = 1.21), anxiety disorders (OR = 1.25), and PTSD (OR = 1.13) [55]. However, other studies demonstrate null associations between musician status and depression symptoms [5] or mixed associations [59]. In *N* = 10,776 Swedish twins, for example, professional and amateur musicians had more self-reported burnout symptoms [54]. However, neither playing music in the past, amateur musicianship, nor professional musicianship was significantly associated with depression or anxiety disorder diagnoses.

Even if musicians are at higher risk, such findings can still be consistent with music-making being beneficial and therapeutic (e.g., depression medication use is elevated in individuals with depressive symptoms *because* it is a treatment). Clinical samples may be useful in disentangling these associations (i.e., examining if those who engage with music more frequently have reduced symptoms), and wider deployment of measures that capture emotion regulation strategies and motivations for engaging with music will help shed light on whether high-risk individuals engage with music in qualitatively different ways than others [51, 57]. Later, we describe how also considering the role of genetic and environmental risk factors in these associations (e.g., if individuals at high genetic and/or environmental risk self-select into music environments because they are therapeutic) can help to clarify these questions.

### Music engagement and externalizing problems

The externalizing domain comprises SUDs, and also includes impulsivity, conduct disorder, and attention-deficit hyperactivity disorder (ADHD), especially in adolescents [20, 24, 60, 61]. Similar to the conclusions for internalizing traits, experimental studies show promising evidence that music engagement interventions may reduce substance use, ADHD, and other externalizing symptoms, but conclusions are limited by methodological limitations. Correlational evidence is sparce, but there is less reason to suspect musicians are at higher risk for externalizing problems.

### Experimental studies

Intervention studies have demonstrated music engagement is helpful in patients with SUDs, including reducing withdrawal symptoms and stress, allowing individuals to experience emotions without craving substance use, and making substance abuse treatment sessions more enjoyable and motivating [62–64] (for a systematic review, see [65]). Similar to the experimental studies of internalizing traits, however, these studies would also benefit from larger samples, better controls, and higher-quality reporting standards.

Music intervention studies for ADHD are of similar quality. Such interventions have been shown to reduce inattention [66], decrease negative mood [67], and increase reading comprehension for those with ADHD [68]. However, there is a great amount of variability among children with ADHD, as some may find music distracting while others may focus better in the presence of music [69].

Little research has been conducted to evaluate music engagement interventions for impulsivity or conduct disorder problems, and findings are mixed. For example, a music therapy study of 251 children showed that beneficial effects on communication skills (after participating in a free improvisation intervention) was significant, though only for the subset of children above age 13 [70]. Another study suggested the promising effects of music therapy on social skills and problem behaviors in 89 students selected based on social/emotional problem behaviors, but did not have a control group [71]. Other smaller studies (*N* < 20 each) show inconsistent results on disruptive behaviors and aggression [72, 73].

### Correlational studies

Correlational studies on externalizing traits are few and far between. A number of studies examined how listening habits for different genres of music relate to more or less substance use [74–77]. However, these studies do not strongly illuminate associations between music engagement and substance use because musical genres are driven by cultural and socioeconomic factors that vary over the lifespan. In the previously cited large study of American electronic medical records [55] where musicianship was associated with more internalizing diagnoses, associations were nonsignificant for “tobacco use disorder” (OR = 0.93), “alcoholism” (OR = 1.01), “alcohol-related disorders” (OR = 1.00), or “substance addiction and disorders” (OR = 1.00). In fact, in sex-stratified analyses, female musicians were at significantly decreased risk for tobacco use disorder (OR = 0.85) [55]. Thus, there is less evidence musicians are at greater risk for externalizing problems than in other areas.

Regarding other aspects of externalizing, some studies demonstrate children with ADHD have poor rhythm skills, opening a possibility that working on rhythm skills may impact ADHD [78, 79]. For example, music might serve as a helpful scaffold (e.g., for attention) due to its regular, predictable rhythmic beat. It will be important to examine whether these associations with music rhythm are also observed for measures of music engagement, especially in larger population studies. Finally, musicians were reported to have lower impulsiveness than prior population samples, but were not compared directly to non-musicians [80, 81].

### Music engagement and thought disorders

Thought disorders typically encompass schizophrenia and bipolar disorder [20]. Trait-level measures include schizotypal symptoms and depression symptoms. Much like internalizing, music interventions appear to provide some benefits to individuals with clinical diagnoses, but musicians may be at higher risk for thought disorders. Limitations of both experimental and correlational studies are similar to those for internalizing and externalizing.

### Experimental studies

Music intervention studies have been conducted with individuals with schizophrenia and bipolar disorder. A recent meta-analysis of 18 music therapy studies for schizophrenia (and similar disorders) [82] demonstrated that music therapy plus standard care (compared to standard care alone) demonstrated improved general mental health, fewer negative symptoms of schizophrenia, and improved social functioning. No effects were observed for general functioning or positive symptoms of schizophrenia. Critiques echoed those described above. Most notably, although almost all studies had low risk of biases due to attrition, unclear risk of bias was evident in the vast majority of studies (>75%) for selection bias, performance bias, detection bias, and reporting bias. These concerns highlight the need for these studies to report more information about their study selection, blinding procedure, and outcomes.

More recent papers suggest similar benefits of music therapies in patients with psychosis [83] and thought disorders [84], with similar limitations (e.g., one study did not include a control group). Finally, although a 2021 review did not uncover more recent articles related to bipolar disorder, they argued that existing work suggests music therapy has the potential both to treat bipolar disorder symptoms and alleviate subthreshold symptoms in early stages of the disorder [85].

### Correlational studies

Much like internalizing, findings from the few existing studies suggest that musicians may be at higher risk for thought disorders. In the large sample of Swedish twins described earlier [54], playing an instrument was associated with more schizotypal symptoms across multiple comparisons (professional musicians vs. non-players; amateur musicians vs. non-players; still plays an instrument vs. never played). However, no associations were observed for schizophrenia or bipolar disorder diagnoses across any set of comparison groups. Another study demonstrated that individuals with higher genetic risk for schizophrenia or bipolar disorder were more likely to be a member of a creative society (i.e., actor or dancer, musician, visual artist, or writer) or work in a profession in these fields [86]. Furthermore, musician status was associated with “bipolar disorder” (OR = 1.18) and “schizophrenia and other psychotic disorders” (OR = 1.18) in US electronic health records (EHRs) [55].

### Interim summary

There is promising evidence that music engagement is associated with better mental health outcomes. Music engagement is positively associated with quality of life, well-being, social connectedness, and emotional competence. However, some individuals who engage with music may be at higher risk for mental health problems, especially internalizing and thought disorders. More research is needed to disentangle these contrasting results, including clarifying how “healthy” music engagement (e.g., for relaxation or social connection) leads to greater well-being or successful emotion regulation, and testing whether some individuals are more likely to use music as a tool to regulate emotions (e.g., those with high neuroticism) [34, 35]. Similarly, it will be important to clarify whether the fact that musicians may be an at-risk group is an extension of working in an artistic field in general (which may feature lower pay or lack of job security) and/or if similar associations are observed with continuous music engagement phenotypes (e.g., hours of practice). As we elaborate on later, genetically informative datasets can help clarify these complex associations, for example by tested whether musicians are at higher *genetic* risk for mental health problems but their music engagement mitigates these risks.

Music intervention studies are feasible and potentially effective at treating symptoms in individuals with clinical diagnoses, including depression, anxiety, and SUDs. However, it will be essential to expand these studies to include larger samples, random sampling, and active control groups that compare the benefits of music interventions to traditional therapies and address possible confounds. These limitations make it hard to quantify how specific factors influence the effectiveness of interventions, such as length/depth of music training, age of sample, confounding variables (e.g., socioeconomic status), and type of intervention (e.g., individual vs. group sessions, song playing vs. songwriting, receptive vs. active methods). Similarly, the tremendous breadth of music engagement activities and measures makes it difficult to identify the specific aspects of music engagement that convey the most benefits to health and well-being [87]. It is therefore necessary to improve reporting quality of studies so researchers can better identify these potential moderators or confounds using systematic approaches (e.g., meta-analyses).

Various mechanisms have been proposed to explain the therapeutic effects of music on mental health, including psychological (e.g., building communities, developing coping strategies) [10, 11] and specific neurobiological drivers (e.g., oxytocin, cortisol, autonomic nervous system activity) [12–14]. However, it will be vital to conduct more systematic research comparing the effects of music interventions to existing therapeutic methods and other types of creative activities (e.g., art [88]) to quantify which effects and mechanisms are specific to music engagement. Music interventions also do not have to be an alternative to other treatments, but may instead support key elements of traditional interventions, such as being engaging, enjoyable, providing social context, and increasing structure and predictability [89]. Indeed, some music therapists incorporate principals from existing psychotherapeutic models [42, 90] and, conversely, newer therapeutic models (e.g., mindfulness) incorporate music into their practice [43, 44]. It is not yet possible to disentangle which aspects of music interventions best synergize with or strengthen standard psychotherapeutic practices (which are also heterogeneous), but this will be possible with better reporting standards and quality experimental design.

To encapsulate and extend these ideas, we next propose a theoretical framework that delineates key aspects of how music engagement may relate to mental health, which is intended to be useful for guiding future investigations in a more systematic way.

### Theoretical framework for future studies

**Fig. 2:**
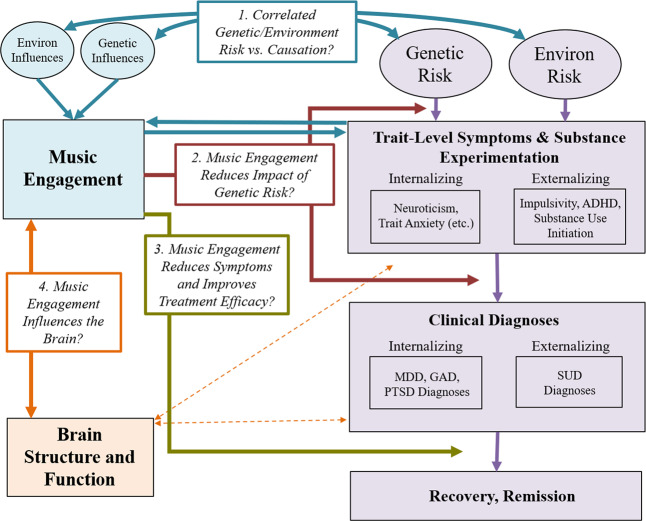
Theoretical model. Progression of mental health problems is based on a diathesis-stress model, where genetic predispositions and environmental exposures result in later problems (which can be remedied through treatment). Potential associations with music engagement include (Path 1; blue arrows) correlated genetic/environmental influences and/or causal associations between music engagement and trait-level mental health outcomes; (Path 2; red arrows) interactions between music engagement and risk factors to predict later trait-level or clinical level symptoms; and (Path 3; gold arrow) direct effects of music engagement on reducing symptoms or improving treatment efficacy. Path 4 (orange arrows) illustrates the importance of understanding how these potential protective associations are driven by neuroanatomy and function. MDD major depressive disorder, GAD generalized anxiety disorder, PTSD posttraumatic stress disorder, SUD substance use disorder(s).

Associations between music engagement and mental health may take multiple forms, driven by several different types of genetic predispositions and environmental effects that give rise to, and interact with, proposed psychological and neurobiological mechanisms described earlier. Figure 2 displays our theoretical model in which potential beneficial associations with music are delineated into testable hypotheses. Four key paths characterize specific ways in which music engagement may relate to (and influence) mental health traits, and thus represent key research questions to be addressed in future studies.

### Path 1: Music engagement relates to mental health through correlated genetic and environmental risk factors and/or causation

The diathesis-stress model of psychiatric disease posits that individuals carry different genetic liabilities for any given disorder [91–93], with disorder onset depending on the amount of negative vs. protective environmental life events and exposures the individual experiences. Although at first glance music engagement appears to be an environmental exposure, it is actually far from it. Twin studies have demonstrated that both music experiences and music ability measures are moderately heritable and genetically correlated with cognitive abilities like non-verbal intelligence [94–97]. Music engagement may be influenced by its own set of environmental influences, potentially including socioeconomic factors and availability of instruments. Thus, music engagement can be viewed as a combination of genetic and environmental predispositions and availability of opportunities for engagement [98] that are necessary to consider when evaluating associations with mental health [54].

**Fig. 3:**
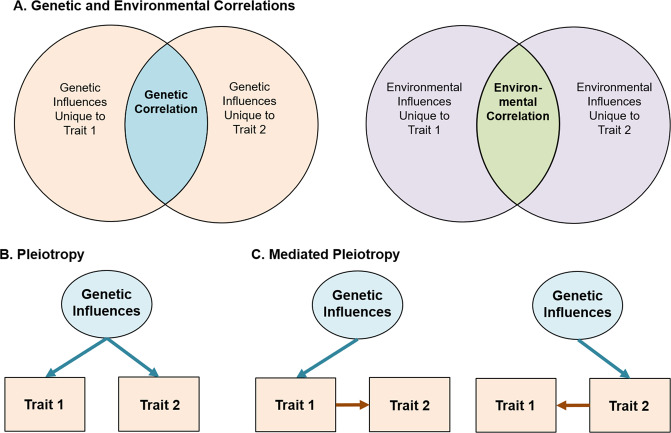
Guide for interpretation of genetic and environmental correlations. Variance in any given trait is explained by a combination of genetic influences (i.e., heritability) and environmental influences. For complex traits (e.g., MDD or depression symptoms), cognitive abilities (e.g., intelligence), and personality traits (e.g., impulsivity), many hundreds or thousands of independent genetic effects are combined together in the total heritability estimate. Similarly, environmental influences typically represent a multitude of factors, from individual life events to specific exposures (e.g., chemicals, etc.). The presence of a genetic or environmental correlation between traits indicates that some set of these influences have an impact on multiple traits. **A** Displayed using a Venn diagram. Identifying the strength of genetic vs. environmental correlations can be useful in testing theoretical models and pave the way for more complex genetic investigations. Beyond this, gene identification efforts (e.g., genome-wide association studies) and additional analyses of the resulting data can be used to classify whether these associations represent specific genetic influences that affect both traits equally (i.e., genetic pleiotropy (**B**)) or whether a genetic influence impacts only one trait which in turn causes changes in the other (i.e., mediated genetic pleiotropy (**C**)). Environmental influences can also act pleiotropically or in a mediated-pleiotropy manner, but only genetic influences are displayed for simplicity.

When examining music-mental health associations, it is thus important to evaluate if associations are in part explained by correlated genetic or environmental influences (see Fig. 3 for schematic and explanation for interpreting genetic/environmental correlations). On one hand, individuals genetically predisposed to engage with music may be at lower risk of experiencing internalizing or externalizing problems. Indeed, music engagement and ability appear associated with cognitive abilities through genetic correlations [3, 99], which may apply to music-mental health associations as well. On the other, individuals at high genetic risk for neuroticism or psychopathology may be more likely to engage with music *because* it is therapeutic, suggesting a genetic correlation in the opposite direction (i.e., increased genetic risk for musicians). To understand and better contextualize the potential therapeutic effects of music engagement, it is necessary to quantify these potential genetic associations, while simultaneously evaluating whether these associations are explained by correlated environmental influences.

Beyond correlated genetic and environmental influences, music engagement and mental health problems may be associated with one another through direct influences (including causal impacts). This is in line with earlier suggestions that music activities (e.g., after-school programs, music practice) engage adolescents, removing opportunities for drug-seeking behaviors [100], increasing their social connections to peers [101], and decreasing loneliness [41]. Reverse causation is also possible, for example, if experiencing mental health problems causes some individuals to seek out music engagement as a treatment. Longitudinal and genetically informative studies can help differentiate correlated risk factors (i.e., genetic/environmental correlations) from causal effects of music engagement (Fig. 2, blue arrows) [102].

### Path 2: Engagement with music reduces the impact of genetic risk

Second, genetic and environmental influences may interact with each other to influence a phenotype. For example, individual differences in music achievement are more pronounced in those who engage in practice or had musically enriched childhood environments [97, 98]. Thus, music exposures may not influence mental health traits directly but could impact the strength of the association between genetic risk factors and the emergence of trait-level symptoms and/or clinical diagnoses. Such associations might manifest as decreased heritability of trait-level symptoms in musicians vs. non-musicians (upper red arrow in Fig. 2). Alternatively, if individuals high in neuroticism use music to help regulate their emotions [34, 35], those who are not exposed to music environments might show stronger associations between neuroticism and later depressive symptoms or diagnoses than those engaged with music (lower red arrow in Fig. 2). Elucidating these possibilities will help disentangle the complex associations between music and mental health and could be used to identify which individuals would benefit most from a music intervention (especially preventative interventions). Later, we describe some specific study designs that can test hypotheses regarding this gene-environment interplay.

### Path 3: Music engagement improves the efficacy of treatment (or acts as a treatment)

For individuals who experience severe problems (e.g., MDD, SUDs), engaging with music may reduce symptoms or improve treatment outcomes. This is the primary goal of most music intervention studies [27, 33] (Fig. 2, gold arrow). However, and this is one of the central messages of this model, it is important to consider interventions in the context of the paths discussed above. For example, if music engagement is genetically correlated with increased risk for internalizing or externalizing problems (Path 1) and/or if individuals at high genetic risk for mental health problems have already been using music engagement to develop strategies to deal with subthreshold symptoms (Path 2), then may be more likely to choose music interventions over other alternatives and find them more successful. Indeed, the beneficial aspects of music training on cognitive abilities appear to be drastically reduced in samples that were randomly sampled [103]. Therefore, along with other necessary reporting standards discussed above [32, 33], it will be useful for studies to report participants’ prior music experience and consider these exposures in evaluating the efficacy of interventions.

### Path 4: Music engagement influences brain structure and function

Exploring associations between music engagement and brain structure and function will be necessary to elucidate the mechanisms driving the three paths outlined above. Indeed, there are strong links between music listening and reward centers of the brain [104, 105] including the nucleus accumbens [106, 107] and ventral tegmental areas [108] that are implicated in the reward system for all drugs of abuse [109–112] and may relate to internalizing problems [113–115]. Moreover, activity in the caudate may simultaneously influence rhythmic sensorimotor synchronization, monetary reward processing, and prosocial behavior [116]. Furthermore, music listening may help individuals control the effect of emotional stimuli on autonomic and physiological responses (e.g., in the hypothalamus) and has been shown to induce the endorphinergic response blocked by naloxone, an opioid antagonist [18, 117].

This work focusing on music listening and reward processing has not been extended to music making (i.e., active music engagement), though some differences in brain structure and plasticity between musicians and non-musicians have been observed for white matter (e.g., greater fractional anisotropy in corpus callosum and superior longitudinal fasciculus) [118–121]. In addition, longitudinal studies have revealed that instrument players show more rapid cortical thickness maturation in prefrontal and parietal areas implicated in emotion and impulse control compared to non-musician children/adolescents [122]. Importantly, because the existing evidence is primarily correlational, these cross-sectional and longitudinal structural differences between musicians and non-musicians could be explained by genetic correlations, effects of music training, or both, making them potentially relevant to multiple paths in our model (Fig. 2). Examining neural correlates of music engagement in more detail will shed light on these possibilities and advance our understanding of the correlates and consequences of music engagement, and the mechanisms that drive the associations discussed above.

### New approaches to studying music and mental health

Using our theoretical model as a guide, we next highlight key avenues of research that will help disentangle these music-mental health associations using state-of-the-art approaches. They include the use of (1) genetic designs, (2) neuroimaging methods, and (3) large biobanks of EHRs.

### Genetic designs

Genetic designs provide a window into the biological underpinnings of music engagement [123]. Understanding the contribution of genetic risk factors is crucial to test causal or mechanistic models regarding potential associations with mental health. At the most basic level, twin and family studies can estimate genetic correlations among music ability or engagement measures and mental health traits or diagnoses. Genetic associations can be examined while simultaneously quantifying environmental correlations, as well as evaluating (bidirectional) causal associations, by testing competing models or averaging across different candidate models [102, 124], informing Path 1.

By leveraging samples with genomic, music engagement, and mental health data, investigators can also examine whether individuals at higher genetic risk for psychopathology (e.g., for MDD) show stronger associations between music engagement measures and their mental health outcomes (Path 2). As a theoretical example, individuals with low genetic risk for MDD are unlikely to have many depressive symptoms regardless of their music engagement, so the association between depressive symptoms and music engagement may be weak if focusing on these individuals. However, individuals at high genetic risk for MDD who engage with music may have fewer symptoms than their non-musician peers (i.e., a stronger negative correlation). This is in line with recent work revealing the heritability of depression is doubled in trauma exposed compared to non-trauma exposed individuals [125].

**Fig. 4:**
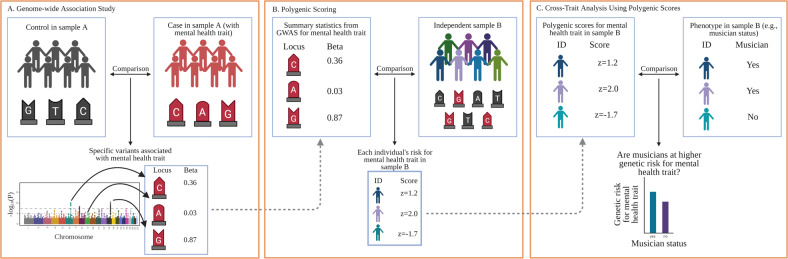
Example of genome-wide association study (GWAS) and polygenic scores. **A** GWAS are conducted by examining whether individual genetic loci (i.e., single-nucleotide polymorphisms, or SNPs, depicted with G, A, C, and T labels within a sample (or meta-analysis) differentiate cases from controls. The example is based on a dichotomous mental health trait (e.g., major depressive disorder diagnosis), but GWAS can be applied to other dichotomous and continuous phenotypes, such as trait anxiety, musician status, or hours of music practice. Importantly, rather than examining associations on a gene-by-gene basis, GWAS identify relevant genetic loci using SNPs from across the *entire genome* (typically depicted using a Manhattan plot, such as that displayed at the bottom of **A**). **B** After a GWAS has been conducted on a given trait, researchers can use the output to generate a polygenic score (sometimes called a polygenic risk score) in any new sample with genetic data by summing the GWAS effect sizes for each SNP allele present in a participant’s genome. An individual with a *z* = 2.0 would have many risk SNPs for that trait, whereas an individual with z = −2 would have much fewer risk SNPs. **C** Once a polygenic score is generated for all participants, it can be applied like any other variable in the new sample. In this example, researchers could examine whether musicians are at higher (or lower) genetic risk for a specific disorder. Other more complex analyses are also possible, such as examining how polygenic scores interact with existing predictors (e.g., trauma exposure) or polygenic scores for other traits to influence a phenotype or predict an intervention outcome. Created with BioRender.com.

Gene–environment interaction studies using *polygenic scores* (i.e., summed indices of genetic risk based on genome-wide association studies; GWAS) are becoming more common [126, 127]. There are already multiple large GWAS of internalizing and externalizing traits [128–130], and the first large-scale GWAS of a music measure indicates that music rhythm is also highly polygenic [131]. Importantly, is not necessary to have all traits measured in the same sample to examine cross-trait relationships. Studies with only music engagement and genetic data, for example, can still examine how polygenic scores for depression predict music engagement, or interact with music engagement measures to predict other study outcomes. Figure 4 displays an example of a GWAS and how it can be used to compute and apply a polygenic score to test cross-trait predictions.

Finally, longitudinal twin and family studies continue to be a promising resource for understanding the etiology and developmental time-course of the correlates of mental health problems. Such designs can be used to examine whether associations between music and mental health are magnified based on other exposures or psychological constructs (gene-by-environment interactions) [132], and whether parents engaged with music are more likely to pass down environments that are protective or hazardous for later mental health (gene-environment correlations) in addition to passing on their genes. These studies also provide opportunities to examine whether these associations change across key developmental periods. The publicly available Adolescent Brain Cognitive Development study, for example, is tracking over 10,000 participants (including twin and sibling pairs) throughout adolescence, with measures of music engagement and exhaustive measures of mental health, cognition, and personality, as well as neuroimaging and genotyping [133, 134]. Although most large samples with genomic data still lack measures of music engagement, key musical phenotypes could be added to existing study protocols (or to similar studies under development) with relatively low participant burden [135]. Musical questionnaires and/or tasks may be much more engaging and enjoyable than other tasks, improving volunteers’ research participation experience.

### Neuroimaging

Another way to orient the design of experiments is through the exploration of neural mechanisms by which music might have an impact on mental health. This is an enormous, growing, and sometimes fraught literature, but there is naturally a great potential to link our understanding of neural underpinnings of music listening and engagement with the literature on neural bases of mental health. These advances can inform the mechanisms driving successful interventions and inform who may benefit the most from such interventions. We focus on two areas among many: (1) the activation of reward circuitry by music and (2) the impact music has on dynamic patterns of neural activity, both of which are likely vectors for the interaction of music and mental health and provide examples of potential interactions.

### Music and reward

The strong effect of music on our emotions has been clearly grounded in its robust activation of reward circuitry in the brain, and motivational and hedonic effects of music listening have been shown to be specifically modulated by dopamine [16, 105, 136]. The prevalence of reward and dopaminergic dysfunction in mental illness makes this a rich area for future studies. For example, emotional responses to music might be used as a substitute for reward circuit deficiencies in depression, and it is intriguing to consider if music listening or music engagement could potentiate such function [137, 138].

### Music and brain network dynamics

The search for neuronally based biomarkers of aspects of mental illness has been a central thrust within the field [139], holding promise for the understanding of heterogeneity within disorders and identification of common mechanistic pathways [140]. A thorough review is beyond the scope of this paper, but several points of contact can be highlighted that might suggest neuro-mechanistic mediators of musical effects on mental health. For example, neurofeedback-directed upregulation of activity in emotion circuitry has been proposed as a therapy for MDD [141]. Given the emotional effects of music, there is potential for using musical stimuli as an adjuvant, or as a more actively patient-controlled output target for neurofeedback. Growing interest in measures of the dynamic complexity of brain activity in health and disease as measured by magnetic resonance imaging or magneto/electroencephalography (M/EEG) [142] provides a second point of contact, with abnormalities in dynamic complexity suggested as indicative of mental illness [143], while music engagement has been suggested to reflect and perhaps affect dynamic complexity [144, 145].

The caveats identified in this review apply equally to such neuro-mechanistic studies [146]. High-quality experimental design (involving appropriate controls and randomized design) has been repeatedly shown to be critical to providing reliable evidence for non-music outcomes of music engagement [103]. For such studies to have maximal impact, analysis of M/EEG activity not at the scalp level, but at the source level, has been shown to improve the power of biomarkers, and their mechanistic interpretability [147, 148]. Moreover, as with genetic influences that typically influence a trait through a multitude of small individual effects [149], the neural underpinnings of music-mental health associations may be highly multivariate. In the longer term, leveraging large-scale studies and large-scale data standardization and aggregation hold the promise of gleaning deeper cross-domain insights, for which current experimentalists can prepare by adopting standards for the documentation, annotation, and storage of data [150].

### Biobanks and electronic health records

Finally, the use of EHR databases can be useful in quantifying associations between music engagement and mental health in large samples. EHR databases can include hundreds of thousands of records and allow for examination with International Statistical Classification of Diseases and Related Health Problems codes, including MDD, SUD, and schizophrenia diagnoses. This would allow for powerful estimates of music-mental health associations, and exploration of music engagement with other health outcomes.

The principal roadblock to this type of research is that extensive music phenotypes are not readily available in EHRs. However, there are multiple ways to bypass this limitation. First, medical records can be scraped using text-mining tools to identify cases of musician-related terms (e.g., “musician”, “guitarist”, “violinist”). For example, the phenome-wide association study described earlier [55] compared musician cases and controls identified in a large EHR database through text-mining of medical records and validated with extensive manual review charts. This study was highly powered to detect associations with internalizing and thought disorders (but showed null or protective effects for musicians for SUDs). Many EHR databases also include genomic data, allowing for integration with genetic models even in the absence of music data (e.g., exploring whether individuals with strong genetic predispositions for musical ability are at elevated or reduced risk for specific health diagnosis).

EHRs could also be used as recruitment tools, allowing researchers to collect additional data for relevant music engagement variables and compare with existing mental health diagnoses without having to conduct their own diagnostic interviews. These systems are not only relevant to individual differences research but could also be used to identify patients for possible enrollment in intervention studies. Furthermore, if recruitment for individual differences or intervention studies is done in patient waiting rooms of specific clinics, researchers can target specific populations of interest, have participants complete some relevant questionnaires while they wait, and be granted access to medical record data without having to conduct medical interviews themselves.

### Concluding remarks

Music engagement, a uniquely human trait which has a powerful impact on our everyday experience, is deeply tied with our social and cultural identities as well as our personality and cognition. The relevance of music engagement to mental health, and its potential use as a therapeutic tool, has been studied for decades, but this research had not yet cohered into a clear picture. Our scoping review and framework integrated across a breadth of smaller literatures (including extant reviews and meta-analyses) relating music engagement to mental health traits and treatment effects, though it was potentially limited due to the lack of systematic literature search or formal quality appraisal of individual studies. Taken together, the current body of literature suggests that music engagement may provide an outlet for individuals who are experiencing internalizing, externalizing, or thought disorder problems, potentially supporting emotion regulation through multiple neurobiological pathways (e.g., reward center activity). Conducting more rigorous experimental intervention studies, improving reporting standards, and harnessing large-scale population-wide data in combination with new genetic analytic methods will help us achieve a better understanding of how music engagement relates to these mental health traits. We have presented a framework that illustrates why it will be vital to consider genetic and environmental risk factors when examining these associations, leading to new avenues for understanding the mechanisms by which music engagement and existing risk factors interact to support mental health and well-being.
